# Is There a Link between Chronic Obstructive Pulmonary Disease and Lung Adenocarcinoma? A Clinico-Pathological and Molecular Study

**DOI:** 10.3390/jpm14080839

**Published:** 2024-08-08

**Authors:** Francesca Lunardi, Giorgia Nardo, Elisabetta Lazzarini, Sofia-Eleni Tzorakoleftheraki, Giovanni Maria Comacchio, Eugenio Fonzi, Michela Tebaldi, Luca Vedovelli, Federica Pezzuto, Francesco Fortarezza, Marco Schiavon, Federico Rea, Stefano Indraccolo, Fiorella Calabrese

**Affiliations:** 1 Department of Cardiac, Thoracic, Vascular Sciences and Public Health, University of Padova, 35128 Padova, Italy; francesca.lunardi@unipd.it (F.L.); giovannimaria.comacchio@aopd.veneto.it (G.M.C.); luca.vedovelli@ubep.unipd.it (L.V.); federica.pezzuto@unipd.it (F.P.); francesco.fortarezza@aopd.veneto.it (F.F.); marco.schiavon@unipd.it (M.S.); federico.rea@unipd.it (F.R.); 2 Basic and Translational Oncology Unit, Istituto Oncologico Veneto IOV—IRCCS, 35128 Padova, Italy; giorgia.nardo@iov.veneto.it (G.N.); elisabetta.lazzarini@iov.veneto.it (E.L.); stefano.indraccolo@unipd.it (S.I.); 3 Department of Pathology, Aristotle University of Thessaloniki, GR-54124 Thessaloniki, Greece; stzorako@auth.gr; 4 IRCCS Istituto Tumori “Dino Amadori” IRST, 47014 Meldola, Italy; eugenio.fonzi@irst.emr.it (E.F.); michela.tebaldi@irst.emr.it (M.T.); 5 Department of Surgery Oncology and Gastroenterology, University of Padova, 35124 Padova, Italy

**Keywords:** COPD, lung adenocarcinoma, morphology, next-generation sequencing

## Abstract

Chronic Obstructive Pulmonary Disease (COPD) and lung cancer are strictly related. To date, it is unknown if COPD-associated cancers are different from the tumors of non-COPD patients. The main goal of the study was to compare the morphological/molecular profiles of lung adenocarcinoma (LUAD) samples of COPD, non-COPD/smokers and non-COPD/non-smokers, and to investigate if a genetic instability also characterized non-pathological areas. This study included 110 patients undergoing surgery for a LUAD, divided into three groups: COPD/smoker LUAD (38), non-COPD/smoker LUAD (54) and non-COPD/non-smoker LUAD (18). The tissue samples were systemically evaluated by pathologists and analyzed using a 30-gene Next Generation Sequencing (NGS) panel. In a subset of patients, tissues taken far from the neoplasia were also included. The non-COPD/smoker LUAD were characterized by a higher proliferative index (*p* = 0.001), while the non-COPD/non-smoker LUAD showed higher percentages of lepidic pattern (*p* = 0.008), lower necrosis, higher fibrosis, and a significantly lower mutation rate in the *KRAS* and *PIK3CA* genes. Interestingly, the same gene mutations were found in pathological and normal areas exclusively in the COPD/smokers and non-COPD/smokers. COPD/smoker LUAD seem to be similar to non-COPD/smoker LUAD, particularly for the genetic background. A less aggressive cancer phenotype was confirmed in non-COPD/non-smokers. The genetic alterations detected in normal lungs from smokers with and without COPD reinforce the importance of screening to detect early neoplastic lesions.

## 1. Introduction

Chronic obstructive pulmonary disease (COPD) and lung cancer are leading causes of morbidity and mortality worldwide. The prevalence of lung cancer in COPD patients is greater than the prevalence of lung cancer in the general population; thus, COPD represents one of the most impactful risk factors for lung carcinoma in smokers, increasing the risk of lung cancer by up to 4.5 folds [1]. COPD and lung cancer share some etiopathogenetic mechanisms, particularly in relation to cigarette smoke [2]. Indeed, tobacco smoke and endogenous oxidants have been shown to induce DNA damage/repair imbalance, a subsequent activation of growth-promoting proto-oncogenes, inactivation of oncosuppressor genes and alteration of cell-death related genes [1]. Immune response (both innate and adaptive) can represent the link between COPD and lung cancer, but further studies are needed. Some authors have shown that immunological background is contributive to excessive oxidative stress leading to lung cancer susceptibility and progression [1,3,4]. Focusing on tobacco smoke, its role in modulating the immune system is complex and controversial, considering that some authors state its relevance to an impaired lung innate immunity [5], while others support the theory of triggered immune responses, suggestive of an autoimmune background for the development and progression of COPD [6]. More recent studies note that a balanced lung microbiome plays a crucial role in the overall effect of host immune response [7] and tobacco smoke causes microbiome dysregulation, thus resulting in persistent inflammation and predisposition to carcinogenesis [8]. Squamous cell carcinoma is the more frequent histotype detected in COPD and/or emphysema patients [9,10] but lung adenocarcinoma (LUAD) has become the most prevalent lung cancer in industrialized countries, with a higher incidence in the baseline GOLD 1 stage patients [11]. It is not completely clarified whether COPD/smoker LUAD patients harbor distinct genomic characteristics compared with non-COPD/smoker LUAD ones, nor their possible prognostic and therapeutic impact. To date, most of the published molecular studies do not precisely distinguish the histotype of non-small cell carcinomas (NSCCs) and report contradictory results. The majority of them highlight a more aggressive nature of COPD-related NSCCs [10,12,13,14,15,16,17,18,19,20,21], while others conclude that there is no impact of COPD on overall survival rates [22,23]. In contrast, some studies report that the immune background of COPD-related cancer is a predictor of a favorable outcome, as the patients are more responsive to immunotherapeutic approaches [24,25,26,27,28]. Only two studies have clearly reported the histotype of the tumors analyzed. In a previous study, our group has shown, in a limited number of cases, that COPD-related LUADs presented morphological and molecular aspects of lower aggressiveness, in terms of prevalent lepidic histological pattern, lower proliferation rate and less frequent *KRAS* mutation [29]. A more recent study concluded that there was no significant difference in the presence of histologic features of LUADs between COPD and non-COPD groups [21]. Considering the limited number of studies about COPD-related LUADs and their contradictory results, the impact of COPD in LUADs is still debated and needs clarification. The hypothesis of the present study is that COPD-related LUAD patients may have a different genetic landscape than LUAD patients without COPD and the main goal was to perform a wide morphological and molecular characterization of LUAD patients, particularly focusing on the most frequent lung cancer-related genes. A secondary goal was to compare the genetic background of tumor tissue samples with “non tumor” specimens, taken far from the neoplasia, to investigate if a genetic instability characterized also non-pathological areas.

## 2. Materials and Methods

### 2.1. Study Design and Population

**Figure 1:**
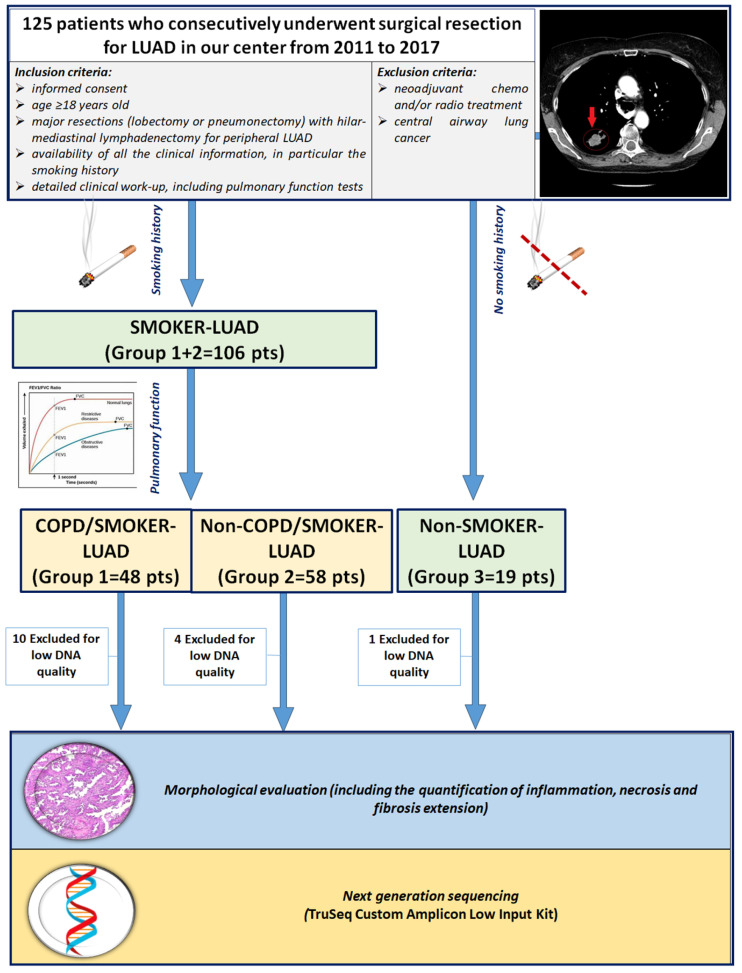
CONSORT diagram. Abbreviations: LUAD: lung adenocarcinoma; COPD: chronic obstructive pulmonary disease; pts: patients.

This retrospective observational study included 125 white patients who consecutively underwent surgical resection for LUAD in our center from 2011 to 2017. A subset of the study population was included in a previous study by our group [29]. Inclusion criteria were (1) age > 18 years old; (2) major resections (lobectomy or pneumonectomy) with hilar-mediastinal lymphadenectomy for peripheral LUAD; (3) no chemo and/or radio neoadjuvant treatment; (4) availability of informed consent; (5) detailed work-up, with complete physical examination, chest radiography, computed tomography scan of the chest, abdomen and brain, bone scan, electrocardiography, routine blood tests and complete pulmonary function tests (PFTs) (including vital capacity—VC; forced expiratory volume in 1 s-FEV1; mid expiratory forced expiratory flow—FEF25–75; forced vital capacity—FVC; total lung capacity—TLC; residual volume—RV; expiratory reserve volume—ERV; and transfer coefficient for carbon monoxide). Patients with extended LUAD, previously chemo and/or radio treated and with central airway lung cancer were excluded in order to avoid distortions of lung architecture or the presence of local and/or systemic inflammatory reactions secondary to oncologic treatment, as previously described [29] (Figure 1). The smoking status of the patients was distinguished as non-smokers (in absence of any history of smoking) and smokers (if the patient was a former or current smoker, of any entity). Patients enrolled in the study were divided into 3 groups according to smoking history and PFTs: COPD/smoker LUAD patients (Group 1: 48 cases), non-COPD/smoker LUAD patients (Group 2: 58 cases) and non-COPD/non-smoker LUAD patients (Group 3: 19 cases). DNA quality was adequate in 110 patients, thus the study focused on those cases. Clinical, anamnestical and morphological data at the time of surgery were recorded in an electronic database. The study was designed in accordance with the Helsinki declaration. Patients gave informed consent for research purposes prior to the surgical procedure.

### 2.2. Morphological Analyses

Immediately after the surgical procedure, lung parenchyma was gently fixed in 10% phosphate-buffered formalin by airway perfusion and widely sampled including both neoplastic and non-neoplastic lung tissues. Endobronchial fixation was carried out to avoid artifactual changes that could affect the evaluation of parenchymal remodeling. After paraffin embedding, sections were cut for routine histological evaluation. The tumors were staged according to the Eighth Edition of Union for International Cancer Control (UICC) TNM Classification of Malignant Tumours [30], and histologically classified according to the 2015 World Health Organization guidelines on the classification of lung cancer [31]. Histological patterns were distinguished and expressed as percentages of lepidic, acinar, papillary, micropapillary and solid. In the tumor sample chosen for molecular investigations (see below), different morphological parameters (inflammation, necrosis and fibrosis) were quantified using a score system from 0 to 3 (0: absent; 1: mild, <10%; 2: moderate, 11–30%; 3: extensive, >30% of the tumor). We mounted 3 μm thick sections of formalin-fixed, paraffin-embedded (FFPE) sections of the tumor were on glass, heated them at 60 °C for 20 min and then processed them using the completely automated Leica Bond III system. Proliferative index was evaluated by Ki-67 immunohistochemistry (MIB-1; Immunotech, France, at a concentration of 1:50), counting at least 100 cells in the most representative areas. Data were expressed as number of positive cells/total cell count %.

### 2.3. DNA Extraction

DNA extraction was performed in one tumor sample for each patient, the same one used for morphological analyses. In a subset of cases (20) DNA was also extracted from one lung sample far from the neoplasia. Five 10 μm sections of tumor area (including a range of 30–80% of neoplastic cells) were collected for each patient. Total DNA extraction was performed using QIAamp DNA FFPE Tissue Kit (Qiagen, Hilden, Germany) according to the manufacturer’s protocol. DNA quantity was evaluated with NanoDrop™ One Spectrophotometer (Thermo Fisher Scientific, Walthman, MA, USA) and for assessing DNA quality, a qualitative Multiplex PCR Assay was performed (Sigma-Aldrich, Merck, Darmstadt, Germany: https://www.sigmaaldrich.com/technical-documents/articles/life-science-innovations/qualitative-multiplex.html, accessed on 1 June 2024).

### 2.4. Next Generation Sequencing

DNA samples were quantified by both Qubit 3.0 Fluorometer (Invitrogen, Carlsbad, CA, USA) and the TruSeq FFPE DNA Library Prep QC Kit (Illumina, San Diego, CA, USA). Targeted next-generation sequencing (NGS) was performed from 40 to 100 ng of DNA per sample, using the TruSeq Custom Amplicon Low Input Kit (Illumina, San Diego, CA, USA), a custom amplicon-based panel targeting over 333 amplicons in 30 lung cancer-related genes (Supplementary Table S1). Libraries were sequenced in 150 bp paired-end reads on a MiSeq Sequencer (Illumina, San Diego, CA, USA).

### 2.5. Bioinformatics Analysis

Raw reads were aligned against the hg19 reference genome using the Miseq Reporter software (v2.4.1), the BAM files were sorted and indexed with SAMtools (v1.3.1) and then the mate-pair information was verified with Picard FixMateInformation (v2.18.27) (VALIDATION_STRINGENCY = SILENT). The BAM files were again indexed with SAMtools. GATK (v3.8-1) was used for local realignment around indels and base quality scores recalibration, to improve the accuracy of the variant calling (IndelRealigner with -rf NotPrimaryAlignment, BaseRecalibrator and PrintReads with -l INFO). Alignment statistics were computed with SAMtools flagstat. The resulting BAM files were pre-processed with SAMtools mpileup (-d 1000000), then analyzed with VarScan2 (v2.3.9) for variant calling, using commands mpileup2snp and mpileup2indel (both with: min-coverage 10, min-var-freq 0.01, strand-filter 0, output-vcf 1), and then filtered as follows:

1. Variants with variant allele frequency (VAF) < 0.05 (5%) were excluded;
2. Variants with coverage < 100X were excluded;
3. Only exonic and splicing variants were kept;
4. Synonymous SNVs were removed;
5. Polymorphisms were excluded, defined as variants having minor allele frequency (MAF) > 0.01 according to Exome Sequencing Project (ESP, https://bio.tools/esp, accessed on 14 April 2023) OR Exome Aggregation Consortium (ExAC, https://ngdc.cncb.ac.cn/databasecommons/database/id/3774, accessed on 14 April 2023) OR 1000 Genomes Project (http://www.internationalgenome.org, accessed on 7 August 2024) OR Genome Aggregation Database (https://gnomad.broadinstitute.org/, accessed on 1 June 2024);
6. We excluded possibly benign variants and variants with uncertain significance according to ClinVar (database update of 20221231, labels considered: ‘Benign’, ‘Benign/Likely benign’, ‘Likely benign’, ‘Uncertain significance’).

As some samples showed an uneven read distribution, we conducted a detailed gene-level coverage analysis to identify possibly false-negative genes (i.e., showing no mutations because of low coverage). The SAMtools depth was used to compute coverage at each genomic position, then ANNOVAR was used to annotate genes and intronic/non-intronic bases. The mean coverage of non-intronic bases was used to determine if a gene had sufficient coverage. A sensitivity analysis (see below) was performed on 7 possible datasets of mutation counts, each using a different threshold of gene coverage (from 100× to 700×). Genes with no mutations that did not meet the threshold were considered as missing values. The dataset with the cut-off at 100X was selected for the subsequent statistical analysis. Bioinformatics analyses and plots were implemented with in-house scripts of bash and Python (v.3.9.7.).

### 2.6. Statistical Analysis

Data are expressed as median (interquartile range) or percentage according to the variable type. For univariate analysis Fisher’s exact test, Pearson’s Chi-squared test or Kruskal–Wallis rank sum test was used. *p*-values were corrected for the false discovery rate according to Benjamini and Hochberg [32]. Missing data were explored with the ‘naniar’ package and patients with more than 9 missing genes data were excluded from subsequent analysis. The resulting dataset was imputed using a random forest method with the function ‘randomForestSRC::impute’. We utilized the Boruta algorithm for feature selection to identify the most important variables able to distinguish COPD patients [33]. Each Boruta iteration involved setting a specific seed and executing the algorithm until stable feature importance was established, with a maximum of 1000 runs per iteration. Post-processing included applying the ‘TentativeRoughFix’ function and extracting the attributes confirmed as important across all iterations. This ensured that only the most consistently significant variables were retained.

## 3. Results

### 3.1. Study Population

**Figure 2:**
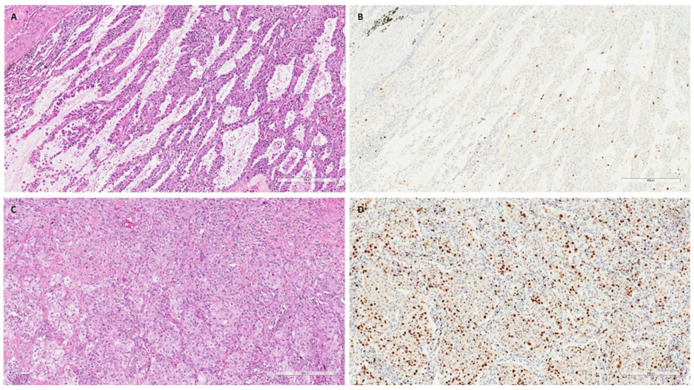
Explanatory images of a non-COPD/non-smoker LUAD and a non-COPD/smoker LUAD. Explanatory images showing a non-COPD/non-smoker LUAD with a lepidic pattern ((**A**), hematoxylin and eosin, scale bar: 300 µm) and a low proliferative index ((**B**), Ki67 immunostaining, scale bar: 300 µm). In contrast, a LUAD with solid pattern ((**C**), hematoxylin and eosin, scale bar: 300 µm) with a high proliferative index ((**D**), Ki67 immunostaining, scale bar: 300 µm) was detected in a non-COPD/smoker patient.

The DNA quality was adequate for the study in 110 patients: 38 COPD/smoker-LUAD, 54 non-COPD/smoker LUAD and 18 non-COPD/non-smoker LUAD patients. The main clinical–anamnestical data from the study population are reported in Table 1. The three groups showed some differences that were expected; in particular, non-COPD/non-smoker LUAD patients were more frequently females (*p* = 0.001) and showed better functional parameters than COPD/smoker LUAD and non-COPD/smoker LUAD ones (*p* < 0.001). Moreover, non-smokers showed lower numbers of blood neutrophils (*p* = 0.046), a lower number of total leucocytes (*p* = 0.012) and lower CRP values (*p* = 0.029) than the other two groups. Interestingly, the non-COPD/smoker LUAD group showed a lower percentage of eosinophils than the COPD/smoker LUAD and non-COPD/non-smoker LUAD groups (*p* = 0.029). The other parameters, including the clinical stage, were not significantly different (Table 1). The COPD/smoker LUAD patients and non-COPD/smoker LUAD patients did not show any differences in their smoking history in terms of pack–years (median, IQR: 40, 27.5–50 vs. 36, 22–50, *p* = 0.245) (Table 1). Among pathological parameters, the LUADs of non-COPD/non-smokers showed a higher median (Q1-Q3) percentage of lepidic patterns than LUADs developed in COPD/smokers and non-COPD/smokers [18 (5–30) vs. 0, 0–5 and 2, 0–24; *p* = 0.008). Non-COPD/smoker LUAD patients were characterized by a higher proliferative index than COPD/smoker LUAD and non-COPD/non-smoker LUAD patients [40 (20–70) vs. 20 (10–58) and 10 (9–21), respectively; *p* = 0.001) (Table 2). Even if not statistically significant, the LUADs of non-COPD/non-smoker patients seem to be characterized by lower percentages of necrosis and higher fibrosis extension (Table 2). All other pathological parameters were not different between the three groups. Explanatory cases are reported in Figure 2.

### 3.2. Distribution of Mutations in COPD, Smokers and Non Smokers Tumor Samples

**Figure 3:**
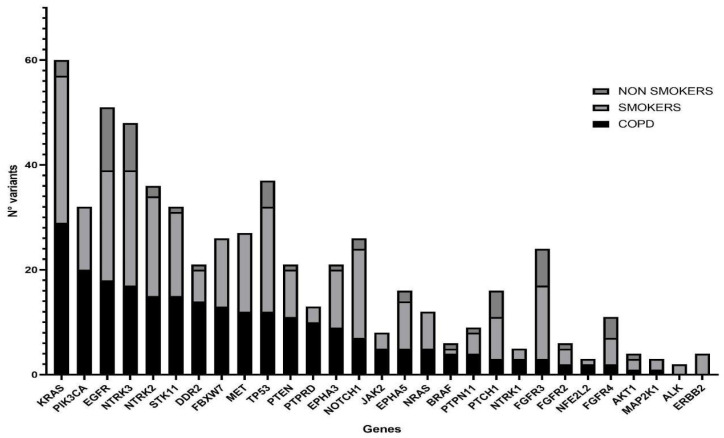
Histogram showing mutated genes in the three study groups. COPD (N = 38), smokers without COPD (N = 54) and non-smokers (N = 18) are represented by filled, light-gray and dark-gray columns, respectively. Genes on the x axis have been ordered according to their mutation rate in the COPD population.

**Figure 4:**
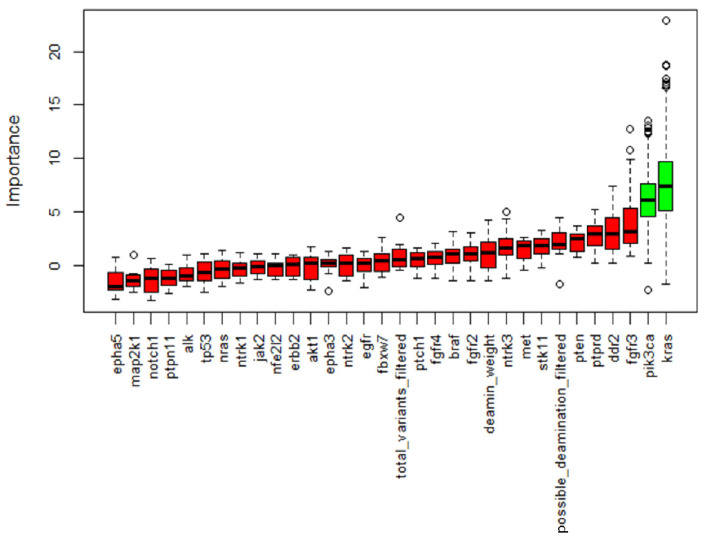
Boruta feature selection output. Important features are marked in green while non-important features are in red. This process was repeated five times and only variables identified in all the five processes were retained. Along with genes, we allowed the algorithm to consider deamination weight, filtered total variants and possible deamination to assess a possible bias in our filtering.

NGS was performed in all samples. After data filtering, a total of 582 sequence variants with VAF > 5% were identified. The samples showed varying proportions of C > T/G > A (possible deamination due to FFPE processing) on the total mutations, but this turned out to be unrelated to the patient condition; therefore, it should not confound the results. Across all samples, the most frequently mutated genes in 110 patients were *KRAS* and *EGFR*, with 60 and 51 variants, respectively, followed by *NTRK3* and *TP53* with 48 and 37 variants (Figure 3). *KRAS* resulted as the most mutated gene in the COPD and smoker groups, with 22 (55%) and 27 (50%) mutated patients, respectively, while among the non-smokers the main mutated gene was *EGFR* with 10 (56%) mutation carriers (Table 3). Statistical analysis showed a significantly lower mutation rate in *KRAS* and *PIK3CA* genes in the non-smokers compared with the other groups, while mutations in all other NGS panel genes were similarly represented (Figure 4).

### 3.3. Analysis of Matched Pathological/Healthy Tissues

A total twenty 20 paired pathological and “healthy” tissue samples were sequenced on the same platform: nine paired samples derived from COPD patients, seven from smoker patients, four from no-smoker patients and the matched data were compared. As detailed in Supplementary Table S2, within the non-smoker group, none of the healthy samples carried pathogenic (P) or likely pathogenic (LP) variants, while within the smoker and the COPD groups, three out of seven (43%) and three out of nine (33%) healthy tissues, respectively, carried P or LP variants. Among smokers, two healthy samples carried mutations different from the matched pathological tissues, while one healthy sample showed the *MET* c.3260-2A > C splice mutation (VAF = 10.2%), shared with the pathological tissue (VAF = 6.3%) that also carried a *PIK3CA* c.1624 G > A p.(Glu542Lys) mutation (VAF = 14.7%). Among COPD patients, two healthy tissues showed different variants from the matched tumor samples, and one carried the *MET* c.3260-2 A > C mutation (VAF = 9.5%), among others, which was also found in the matched pathological tissue with a similar VAF (8.2%).

## 4. Discussion

COPD and lung cancer share many important features, such as their epidemiology, the main clinical features, a high mortality rate and a significant public health impact worldwide. COPD patients are at increased risk of NSCC and patients with both COPD and NSCC are characterized by a worse outcome [13,14,16,34,35,36,37,38].

While the exact underlying mechanisms have not yet been clarified, the major potential contributing factors include genomic, immune and microenvironment dysregulation [39]. It is known that the concomitant presence of different diseases in individual patients may strongly influence the carcinogenic process, and consequently, the morphological/molecular lung cancer phenotype [29]. Thus, to the best of our knowledge this is the first study that comprehensively studied COPD patients with a LUAD from a clinical, morphological and genetic point of view, comparing them with a LUAD developed in smokers and non-smoker patients without COPD.

In a previous study by our group, COPD/smoker LUAD showed the molecular and morphological features of lower aggressiveness compared with smokers, in particular a reduced solid pattern, lower cell proliferation, and less frequent *KRAS* mutations [29].

The present study confirmed the presence of a higher proliferative index in non-COPD/smoker LUAD compared with COPD/smoker LUAD (median, Q1–Q3: 40, 20–70 vs. 20, 10–58), thus supporting a more aggressive cancer phenotype.

Moreover, as expected, non-COPD/non-smoker LUAD patients showed a higher prevalence of lepidic pattern and lower proliferative index in comparison to the other two groups, and this result is in line with the better prognosis of LUADs developed in non-smokers [40,41].

Among the other morphological features, a tendency towards less necrosis and more fibrosis seems to characterize LUADs developed in non-smokers, potentially supporting the less aggressive phenotype, even if statistical significance was not reached. In recent decades, the prognostic significance of necrosis in resectable NSCC has been demonstrated by many investigators [42]. Also, fibrotic extension has been hypothesized as a good prognostic factor in LUAD settings, even if most studies are in the neoadjuvant setting or only focusing on central fibrosis [43].

These data need to be validated using a morphometrical quantitative analysis of inflammation, fibrosis and necrosis. Moreover, a specific characterization of inflammatory cell infiltration using artificial intelligence approaches could be crucial to highlight differences between these groups and to identify the most crucial inflammatory cell actors.

Considering genetic background, one study on COPD and lung cancer showed that many candidate gene loci overlap in these two diseases, with significant associations between multiple chromosomal loci and single nucleotide polymorphisms (SNPs) [44]. Moreover, even epigenetic factors seem to be instrumental in the occurrence of COPD and lung cancer, as oxidative stress and inflammatory responses change the redox potential of cells due to unstable genomes and ultimately epigenetic modification [44].

In our study, NGS analysis highlighted higher abundance of *KRAS* and *PIK3CA* mutations in COPD/smoker and non-COPD/smoker LUAD, compared with the non-smoker population. *KRAS* mutations are typically smoke associated in lung cancer and their lower prevalence in the non-smoker population observed in our study is thus expected. Most of these *KRAS* mutations (>85%) were found in classical mutational hotspots, both in the COPD and the non-COPD/smoker LUAD groups, also without any difference for the type of KRAS mutations.

The lipid kinase phosphatidylinositol-3 kinase (PI3K) family is involved in a number of normal cellular processes. For example, it plays an important role in the activation of serine/threonine kinase AKT, which activates a lot of downstream factors such as mTOR [45]. *PIK3CA* mutations have been found in a large variety of human tumors, and a frequency of 2–7% in NSCC was observed according to previous studies. In particular, it was shown to be associated with smoking history and hypothesized to be a prognostic factor in this context [46], whereas the actionability of *PIK3CA* mutations in NSCC remains undetermined.

With regard to the analysis of histologically normal tissue, our findings are in line with previous studies and support the “field cancerization” model, originally described in oral stratified squamous epithelium [47] and subsequently extended to other tissues exposed to carcinogens, such as the lung [48]. In fact, these mutations were found exclusively in the COPD and non-COPD/smoker LUAD groups and not in the non-COPD/non-smoker group. Most of the observed variants are represented by G > A/T > C transitions, so we can hypothesize that at least a proportion of them can be due to deamination effects. However, fixation artifacts are reported by various studies as detected at lower frequencies (VAF < 5%) [49], compared with the variants described in our study. Altogether, our findings might underscore the existence of clones in the process of transforming into tumor cells, which at the time of tissue analysis accumulated some but not all the genetic alterations required to start the neoplastic process. The genetic alterations detected in normal lungs from smoker patients with and without COPD reinforce the importance of refining screening campaigns to detect early neoplastic lesions, particularly those based on low-dose computed tomography, that have been associated with lower mortality in high-risk populations [50].

The main limitation of the study is the low number of COPD/smokers compared with non-COPD/smokers, thus the validation of these results in a wider case series, including patients from other centers, is mandatory. Moreover, another limitation is related to the use of morphometrical/artificial intelligence approaches, which will be necessary for a better characterization of LUADs developed in COPD and non-COPD patients. Finally, the amount of smoking was not considered in the analyses; thus, further studies are needed to explore this aspect.

Consequently, this knowledge would be of considerable help in the fight against lung cancer both for individualized follow-up and from therapeutic perspectives, providing a rationale to develop targeted and more effective treatments.

## Figures and Tables

**Table 1:** Major clinical data.

Characteristics	COPD/Smoker LUAD N = 38	Non-COPD/Smoker LUAD N = 54	Non-COPD/Non-Smoker LUAD N = 18	*p*-Value ^a^	q-Value ^b^
Sex				0.001	0.015
males	30 (79%)	34 (63%)	5 (28%)		
females	8 (21%)	20 (37%)	13 (72%)		
Age	72 (68, 75)	69 (62, 73)	65 (59, 72)	0.11	0.3
GOLD stage				>0.9	>0.9
I	18 (47.5%)	0 (NA%)	0 (NA%)		
II	18 (47.5%)	0 (NA%)	0 (NA%)		
III	2 (5%)	0 (NA%)	0 (NA%)		
Smoking history (pack years)	40 (27.5–50)	36 (22–50)	-	0.245	0.67
FEV1 (% of predict)	76 (64, 87)	93 (88, 113)	117 (104, 130)	<0.001	<0.001
FEV1/FV (% of predict)	67 (58, 70)	83 (78, 85)	82 (78, 86)	<0.001	<0.001
FVC (% of predict)	92 (74, 101)	92 (82, 105)	119 (108, 128)	<0.001	0.002
DLCO/VA (%)	70 (48, 89)	80 (65, 91)	84 (81, 99)	0.085	0.2
SUV	6 (2, 11)	6 (2, 12)	2 (1, 5)	0.061	0.2
WBC (n × 10^9^/L)	7.35 (5.78, 8.68)	6.92 (5.45, 9.30)	5.52 (4.70, 7.01)	0.012	0.072
RBC (n × 10^12^/L)	4.50 (4.18, 5.00)	4.42 (4.14, 4.77)	4.62 (4.21, 4.96)	0.7	0.8
HgB (g/dL)	13.70 (12.35, 14.65)	13.70 (12.50, 14.70)	13.80 (13.03, 14.80)	0.7	0.8
Neutrophils (n × 10^9^/L)	4.40 (3.54, 5.74)	4.02 (3.06, 6.02)	2.87 (2.81, 4.52)	0.046	0.2
Neutrophils (%)	64 (56, 69)	62 (56, 69)	56 (52, 66)	0.3	0.4
Lymphocytes (n × 10^9^/L)	1.77 (1.48, 2.12)	1.75 (1.55, 2.06)	1.62 (1.39, 1.93)	0.7	0.8
Lymphocytes (%)	24 (20, 32)	26 (20, 32)	31 (24, 33)	0.3	0.4
Monocytes (n × 10^9^/L)	0.60 (0.49, 0.70)	0.57 (0.42, 0.70)	0.49 (0.40, 0.58)	0.082	0.2
Monocytes (%)	8.55 (7.03, 10.05)	8.00 (7.10, 9.80)	9.30 (6.90, 10.50)	0.7	0.8
Eosinophils (n × 10^9^/L)	0.16 (0.06, 0.26)	0.08 (0.05, 0.16)	0.12 (0.06, 0.26)	0.079	0.2
Eosinophils (%)	2.05 (1.33, 3.63)	1.30 (0.80, 2.30)	2.20 (1.30, 3.80)	0.029	0.13
Basophils (n × 10^9^/L)	0.030 (0.020, 0.040)	0.020 (0.010, 0.030)	0.020 (0.020, 0.030)	0.13	0.3
Basophils (%)	0.40 (0.30, 0.60)	0.30 (0.20, 0.50)	0.40 (0.30, 0.60)	0.2	0.3
ESR (mm/h)	21 (12, 32)	20 (10, 28)	14 (9, 20)	0.090	0.2
CRP (mg/L)	2.0 (1.4, 6.9)	2.9 (1.3, 4.7)	0.8 (0.3, 2.9)	0.029	0.13
Clinical stage				0.2	0.3
IA	7 (18.5%)	17 (31.5%)	6 (33%)		
IB	16 (42%)	11 (20.5%)	9 (50%)		
IIA	4 (10.5%)	3 (6%)	0 (0%)		
IIB	7 (18.5%)	10 (18%)	2 (11%)		
IIIA	4 (10.5%)	10 (18%)	1 (6%)		
IIIB	0 (0%)	3 (6%)	0 (0%)		

Abbreviations: CRP: C reactive protein; DLCO: diffusing lung capacity; ESR: erythrocyte sedimentation rate; FEV1: forced expiratory volume in the first second; FVC: forced vital capacity; HgB: hemoglobin; RBC: red blood cells; SUV: standard uptake volume; VA: alveolar volume; VC: vital capacity; WBC: white blood cells. Categorical data are expressed as n (%); continuous variables are expressed as median (IQR). ^a^ Pearson’s Chi-squared test; Kruskal–Wallis rank sum test; Fisher’s exact test. ^b^ False discovery rate correction for multiple testing.

**Table 2:** Major pathological data.

Characteristic	COPD/Smoker LUAD N = 38	Non-COPD/Smoker LUAD N = 54	Non-COPD/Non-Smoker LUAD N = 18	*p*-Value ^a^	q-Value ^b^
Tumor cells (%)	70 (50, 80)	65 (50, 80)	70 (70, 80)	0.5	0.7
Prevalent pattern				0.3	0.4
Lepidic pattern	1 (2.5%)	8 (15%)	1 (5.6%)		
Acinar pattern	28 (74%)	32 (59%)	14 (78%)		
Papillary pattern	1 (2.5%)	4 (7.5%)	0 (0%)		
Solid pattern	8 (21%)	10 (18.5%)	3 (17%)		
Lepidic pattern (%)	0 (0, 5)	2 (0, 24)	18 (5, 30)	0.008	0.057
Acinar pattern (%)	60 (42, 85)	50 (20, 75)	60 (45, 84)	0.14	0.3
Papillary pattern (%)	0 (0, 0)	0 (0, 8)	0 (0, 8)	0.7	0.8
Micropapillary pattern (%)	0 (0, 2)	0 (0, 1)	0 (0, 4)	>0.9	>0.9
Solid pattern (%)	10 (0, 35)	0 (0, 32)	0 (0, 0)	0.2	0.3
WHO Grading					
1	1 (2%)	5 (9%)	1 (5%)		
2	17 (45%)	21 (39%)	14 (78%)		
3	20 (53%)	28 (52%)	3 (17%)		
MIB1 (%)	20 (10, 58)	40 (20, 70)	10 (9, 21)	0.001	0.011
Necrosis (%)				0.2	0.3
0	6 (16%)	12 (22%)	7 (39%)		
≤10%	19 (50%)	25 (46%)	7 (39%)		
11–30%	4 (10%)	7 (13%)	4 (22%)		
>30%	9 (24%)	10 (19%)	0 (0%)		
Inflammation (%)				0.2	0.3
0	0 (0%)	0 (0%)	1 (6%)		
≤10%	13 (34%)	24 (44%)	4 (22%)		
11–30%	18 (47.5%)	23 (43%)	11 (61%)		
>30%	7 (18.5%)	7 (13%)	2 (11%)		
Fibrosis (%)				0.2	0.3
0	3 (8%)	4 (7%)	0 (0%)		
≤10%	16 (42%)	22 (41%)	3 (17%)		
11–30%	12 (32%)	18 (33%)	8 (44%)		
>30%	7 (18%)	10 (19%)	7 (39%)		
Vascular invasion				0.5	0.7
No	18 (47%)	23 (43%)	9 (50%)		
Yes	20 (53%)	31 (57%)	9 (50%)		
Pleural invasion				0.6	0.8
No	20 (53%)	27 (50%)	11 (61%)		
Yes	18 (47%)	27 (50%)	7 (39%)		
Type of visceral pleura invasion				>0.9	>0.9
PL0	20 (53%)	27 (50%)	11 (61%)		
PL1	15 (39%)	23 (43%)	6 (33%)		
PL2	3 (8%)	4 (7%)	1 (6%)		
Perineural invasion				0.5	0.7
No	34 (89%)	50 (93%)	18 (100%)		
Yes	4 (11%)	4 (7%)	0 (0%)		
Lymph node invasion				0.089	0.2
No	30 (79%)	38 (70%)	17 (94%)		
Yes	8 (21%)	16 (30%)	1 (6%)		
Type of lymph node invasion				0.14	0.3
0	30 (79%)	38 (70%)	17 (94%)		
1	6 (16%)	7 (13%)	1 (6%)		
2	2 (5%)	9 (17%)	0 (0%)		

Abbreviations: COPD: Chronic Obstructive Pulmonary Disease. Categorical data are expressed as n (%); continuous variables are expressed as median (IQR). ^a^ Pearson’s Chi-squared test; Kruskal–Wallis rank sum test; Fisher’s exact test. ^b^ False discovery rate correction for multiple tests.

**Table 3:** Prevalence of mutated patients within the three study groups.

Gene	COPD/Smoker LUAD N = 38	Non-COPD/Smoker LUAD N = 54	Non-COPD/Non-Smoker LUAD N = 18
*KRAS*	21 (55%)	27 (50%)	3 (17%)
*EGFR*	9 (24%)	12 (22%)	10 (56%)
*NTRK3*	8 (21%)	14 (26%)	5 (28%)
*TP53*	10 (26%)	15 (28%)	4 (22%)
*NTRK2*	8 (21%)	10 (19%)	1 (6%)
*PIK3CA*	7 (18%)	9 (17%)	0 (0%)
*STK11*	9 (24%)	13 (24%)	1 (6%)
*MET*	5 (13%)	10 (19%)	0 (0%)
*NOTCH1*	6 (16%)	11 (20%)	2 (11%)
*FBXW7*	5 (13%)	5 (9%)	0 (0%)

Patients with at least one somatic variant in the ten mainly mutated genes are shown.

## Data Availability

The raw data supporting the conclusions of this article will be made available by the authors on request.

## Supplementary Materials

The following supporting information can be downloaded at: https://www.mdpi.com/article/10.3390/jpm14080839/s1, Table S1: Genes included in the custom lung cancer NGS panel (designed on hg19 genome); Table S2: Mutational analysis of paired pathological and “healthy” tissue samples.
