# Supplementary material for: Is There a Link between Chronic Obstructive Pulmonary Disease and Lung Adenocarcinoma? A Clinico-Pathological and Molecular Study
Source: J Pers Med. 2024 Aug 8;14(8):839. doi: 10.3390/jpm14080839 (PMC11355616; doi:10.3390/jpm14080839)
Supplement: Supplementary file 1 [file jpm-14-00839-s001.zip › jpm-3097428-supplementary.pdf]

Supplementary table S1. Genes included in the custom lung cancer NGS panel (designed on hg19 genome)

AKT1	*FGFR2*	*NTRK1*
ALK	*FGFR3*	*NTRK2*
BRAF	*FGFR4*	*NTRK3*
DDR2	*JAK2*	*PIK3CA*
EGFR	*KRAS*	*PTCH1*
EPHA3	*MAP2K1*	*PTEN*
EPHA5	*MET*	*PTPN11*
ERBB2	*NOTCH1*	*PTPRD*
FBXW7	*NRAS*	*STK11*
FGFR1	*NRF2*	*TP53*

Supplementary table S2. Mutational analysis of paired pathological and "healthy" tissue samples. Only pathogenic/likely pathogenic genetic variants are shown (P pathological tissue, H healthy tissue, S smokers, NS non smokers). Variants shared by the matched healthy and pathological tissue are depicted in bold.

Sample_ID	Group	Tissue	Pathogenic/likely pathogenic variants	VAF
C434/11	COPD	P	-	
C434/11A		H	-	
C843/11	COPD	P	*KRAS*:NM_004985:exon2:c.G34T:p.G12C	22.3
C843/11A		H	-	
C766/11	COPD	P	-	
C768/11		H	-	
15-I-987 H1	COPD	P	*TP53*:NM_000546:exon4:c.G158A:p.W53X	16.32
			STK11:NM_000455:exon2:c.291-1G>A	6.73
			PIK3CA:NM_006218:exon2:c.G278A:p.R93Q	7.82
			EPHA:NM_005233:exon5:c.971-1G>A	5.01
			FBXW7:NM_018315:exon8:c.G1028A:p.G343E	10.27
			***MET*:NM_000245:exon16:c.3260-2A>C**	8,22

15-I-987 H7		H	*ERBB2*:NM_001289937:exon20:c.C2458T:p.Q820X	5.9
			TP53:NM_000546:exon10:c.994-1G>A;	7.8
			TP53:NM_000546:exon6:c.C667T:p.P223S	7.24
			***MET*:NM_000245:exon16:c.3260-2A>C**	9.5
			NOTCH1:NM_017617:exon34:c.C6673T:p.Q2225X	6.25
			NTRK2:NM_006180:exon4:c.69delG:p.V23fs	6.04
15-I-1288 D1	COPD	P	*MET*:NM_000245:exon2:c.C1042T:p.Q348X	10.9
15-I-1288 D4		H	-	
15-I-1239 C3	COPD	P	*KRAS*:NM_004985:exon3:c.290+2T>C	6.12
			TP53:NM_000546:exon10:c.1083delG:p.G361fs	5.33
			STK11:NM_000455:exon1:c.3delG:p.M1fs	6.78
			EGFR:NM_005228:exon18:c.G2171A:p.G724D	7.4
			PTPRD:NM_001171025:exon8:c.1055delA:p.N352fs	5.36
			NTRK2:NM_006180:exon19:c.2172+1G>A	12.33
15-I-1239 C5		H	*MET*:NM_000245:exon16:c.3260-2A>C	11.15
16 M 0344	COPD	P	-	
16 I 0827 A8		H	-	
16 M 433	COPD	P	*KRAS*:NM_004985:exon2:c.G35A:p.G12D	18.19
			PIK3CA:NM_006218:exon10:c.G1633A:p.E545K	31.48
16 I 1141 A8		H	-	
15-30290 04	COPD	P	*KRAS*:NM_004985:exon2:c.G35C:p.G12A	16.71
15-30290		H	*FGFR3*:NM_000142:exon7:c.G836A:p.S279N	5.64
C52/12	S	P	*BRAF*:NM_004333:exon15:c.T1799A:p.V600E	8.08
			EGFR:NM_005228:exon18:c.G2155T:p.G719C	7.56
C52/12A		H	-	
15-I-1130 A2	S	P	-	
15-I-1590 E2		H	-	
15-M-0987	S	P	*EGFR*:NM_005228:exon20:c.G2303T:p.S768I	17.57
			EGFR:NM_005228:exon18:c.G2156C:p.G719A	41.56
			TP53:NM_000546:exon5:c.461delG:p.G154fs	41.09
15-I-1446 J1		H	-	
15-M-1175	S	P	*KRAS*:NM_004985:exon2:c.G35C:p.G12A	15.4
15-I-1804 I5		H	-	
15-I-941 B2	S	P	*DDR2*:NM_006182:exon13:c.G1715A:p.G572E	6.39
			TP53:NM_000546:exon7:c.A701G:p.Y234C	33.13
			STK11:NM_000455:exon3:c.G388A:p.E130K	15.03
			PIK3CA:NM_006218:exon21:c.C2977T:p.Q993X	5.28
			FBXW7:NM_018315:exon8:c.G1105T:p.E369X	18.46
			MET:NM_000245:exon19:c.T3778G:p.F1260V	7.67
			NTRK2:NM_006180:exon20:c.G2281A:p.E761K	7.72
15-I-941 B4		H	*KRAS*:NM_004985:exon3:c.C208T:p.Q70X	8.33
15-I-1112 I3	S	P	*PIK3CA*:NM_006218:exon10:c.G1624A:p.E542K	14.7
			***MET*:NM_000245:exon16:c.3260-2A>C**	6,3
15-I-1112 I5		H	***MET*:NM_000245:exon16:c.3260-2A>C**	10,2
15-I-1276 A3	S	P	*KRAS*:NM_004985:exon2:c.G35T:p.G12V	33.57
			PIK3CA:NM_006218:exon2:c.335delT:p.I112fs	5.57
			EPHA3:NM_005233:exon2:c.C115T:p.Q39X	5.93
			MET:NM_000245:exon19:c.G3746T:p.W1249L	5.18
15-I-1276 A5		H	*PIK3CA*:NM_006218:exon10:c.G1656A:p.W552X	7.36

			MET:NM_000245:exon16:c.3260-2A>C	8.53
15 M 1292	NS	P	*KRAS*:NM_004985:exon2:c.G35T:p.G12V	26.59
16 I 2061 I7		H	-	
C919/11	NS	P	*BRAF*:NM_004333:exon15:c.T1799A:p.V600E	20.94
C5/12		H	-	
C780/11	NS	P	-	
C782/11		H	-	
15-I-0997 F3	NS	P	*DDR2*:NM_006182:exon13:c.G1708A:p.G570R	7.82
			PTPN11:NM_002834:exon13:c.C1498T:p.Q500X	6.15
			KRAS:NM_004985:exon3:c.G223A:p.G75R	14.2
			TP53:NM_000546:exon7:c.G713T:p.C238F	38.08
			TP53:NM_000546:exon6:c.T617A:p.L206X	9.83
15-I-997 F4		H	-	
